# Short term effects of a low-carbohydrate diet in overweight and obese subjects with low HDL-C levels

**DOI:** 10.1186/1472-6823-10-18

**Published:** 2010-11-09

**Authors:** Ahmet Selçuk Can, Canan Uysal, K Erhan Palaoğlu

**Affiliations:** 1 Department of Medicine, Kadir Has University, Faculty of Medicine, Vefa Bey Sokak, No: 5, 34349, Gayrettepe, Beşiktaş, Istanbul, Turkey; 2 Medical Nutrition Center, Sezai Selek Sokak, No: 14/11, Nişantaşı, Şişli, Istanbul, Turkey; 3 Department of Biochemistry, Vehbi Koç Foundation American Hospital, Güzelbahçe Sokak, No: 20, Nişantaşı, Şişli, Istanbul, Turkey

## Abstract

**Background:**

The aim of this study was to evaluate short-term effects of a low-carbohydrate diet in overweight and obese subjects with low HDL-C levels.

**Methods:**

Overweight (BMI between 25-30 kg/m^2^) or obese (BMI over 30 kg/m^2^) subjects with low HDL-C levels (men with HDL-C <1.03, women <1.29 mmol/l) were invited to the study. A 1400 kcal 75-gram carbohydrate (CHO) diet was given to women and an 1800 kcal 100-gram CHO diet was given to men for four weeks. The distribution of daily energy of the prescribed diet was 21-22% from CHO, 26-29% from protein and 49-53% from fat. Subjects completed a three-day dietary intake record before each visit. Anthropometric indices, body fat ratio, blood lipids, glucose and insulin were measured. Baseline and week-four results were compared with a Wilcoxon signed ranks test.

**Results:**

Twenty-five women and 18 men participated. Basal median LDL-C level of men was 3.11 and basal median LDL-C level of women was 3.00 mmol/l. After four weeks of a low-carbohydrate diet, the median energy intake decreased from 1901 to 1307 kcal/day, daily energy from carbohydrate from 55% to 33%, body weight from 87.7 to 83.0 kg and HDL-C increased from 0.83 to 0.96 mmol/l in men (p < 0.002, for all). After four weeks of a low-carbohydrate diet, the median energy intake tended to decrease (from 1463 to 1243 kcal, p = 0.052), daily energy from carbohydrate decreased from 53% to 30% (p < 0.001) and body weight decreased from 73.2 to 70.8 kg (p < 0.001) in women, but HDL-C did not significantly change (from 1.03 to 1.01 mmol/l, p = 0.165). There were significant decreases in body mass index, waist circumference, body fat ratio, systolic blood pressure, total cholesterol, triglyceride and insulin levels in all subjects.

**Conclusions:**

HDL-C levels increased significantly with energy restriction, carbohydrate restriction and weight loss in men. HDL-C levels didn't change in women in whom there was no significant energy restriction but a significant carbohydrate restriction and a relatively small but significant weight loss. Our results suggest that both energy and carbohydrate restriction should be considered in overweight and obese subjects with low HDL-C levels, especially when LDL-C levels are not elevated.

## Background

A low high density lipoprotein cholesterol (HDL-C) level is an independent risk factor for atherosclerotic cardiovascular disease (CVD) [1]. Even in subjects with a low density lipoprotein cholesterol (LDL-C) level that is below 60 mg/dl, either achieved with lipid lowering agents or occurring spontaneously, HDL-C levels show an inverse relationship with CVD risk and a U-shaped relationship with all-cause mortality [2]. The increase in HDL-C level has been found to have a strong independent effect in reducing CVD risk after adjustment of other lipid changes in patients who are on lipid lowering therapy [3]. Although relatively effective LDL-C lowering medical therapies are available, drugs that raise HDL-C level as the principal pharmacodynamic effect do not exist. Physical activity, cessation of smoking, weight loss, moderate alcohol intake, a diet rich in omega-3 polyunsaturated fatty acids and a diet low in carbohydrates are HDL-C raising strategies [4]. The Mediterranean diet has beneficial effects on CVD risk [5] and is effective in raising HDL-C levels [6]. A targeted lifestyle change program improves CVD risk factors and the distribution of plasma lipoprotein subclasses, especially small dense LDL-C particles [7]. The United States National Cholesterol Education Program Adult Treatment Panel III (NCEP) recommends therapeutic lifestyle changes diet to reduce the risk of CVD [1]. The distribution of daily energy intake is 50-60% from carbohydrate, 15% from protein and 25-35% from fat in the therapeutic lifestyle changes diet. American Heart Association and World Health Organization also recommend a low-fat diet [8,9]. In view of the recent data on the benefits of low-carbohydrate diets, these official recommendations have been challenged [10-12]. The primary objective of the therapeutic lifestyle changes diet is to reduce LDL-C levels to prevent CVD, but low-fat diets decrease HDL-C levels and increase triglycerides as well [13-18]. There are no dietary intervention studies performed in subjects with only low HDL-C levels. The aim of this study was to evaluate the effects of a low-carbohydrate diet in overweight and obese subjects with low HDL-C levels.

## Methods

Subjects were invited to the study from the Turkish Heart Study database. Turkish Heart Study is an epidemiological survey of CVD risk factors periodically performed in Turkey. In 2005 and 2006, we reviewed our 2003 Turkish Heart Study data [19] and invited overweight [body mass index (BMI) ≥25 and <30 kg/m^2^] and obese (BMI ≥30 kg/m^2^) subjects with a low HDL-C level. NCEP HDL-C criterion [HDL-C <50 mg/dl (1.29 mmol/l) for women and <40 mg/dl (1.03 mmol/l) for men] was used to categorize subjects to low HDL-C status [1]. The exclusion criteria were the presence of a major medical illness like renal disease, liver disease, cancer, diabetes and CVD and the use of lipid-lowering drugs. A phone call was made to subjects for invitation and to explain the study. Subjects who agreed to participate were instructed on how to record dietary intake and were asked to come to our outpatient offices between 7 am and 10 am after a 10-hour fast. At each visit blood pressure, anthropometric indices and body fat ratio were measured and a fasting blood sample was obtained. The study was approved by the Ethics Committee of Kadir Has University, Faculty of Medicine. All subjects signed written informed consent.

### Clinical variables

Body weight and composition were measured with a Tanita Body Composition Analyzer BC-418 MA (Tanita Corporation, Tokyo, Japan). Bioimpedance analysis was performed in the morning before breakfast and subjects were instructed to void before the measurement. Body weight was measured in kilograms to within 0.1 kg. Height was measured to within 0.5 cm with a measuring stick. BMI was calculated by dividing the weight in kilograms to squared height in meters. Waist circumference was measured at the midpoint between lower margin of the rib cage and superior iliac crest during mild expiration with a non-elastic measuring tape. Waist circumference was measured to the nearest 0.5 cm. All measurements were taken when subjects were on light clothing and after shoes were taken off. Blood pressure was measured on the right arm with an automated sphygmomanometer (Omron automatic blood pressure monitor with IntelliSense^®^, Bannockburn, IL, USA) after fifteen minute of rest with the subject in the sitting position. The mean of two recordings, five minutes apart was recorded.

The metabolic syndrome was defined by the NCEP criteria that were modified by the American Heart Association and the United States National Heart Lung and Blood Institute [1,20]. According to NCEP, metabolic syndrome is diagnosed if three of the following five components are abnormal: 1) waist circumference≥102 cm in men or ≥88 cm in women, 2) systolic blood pressure≥130 mmHg or diastolic blood pressure≥85 mmHg, 3) serum triglycerides≥150 mg/dl (1.69 mmol/l), 4) HDL-C <50 mg/dl (1.29 mmol/l) for women and <40 mg/dl (1.03 mmol/l) for men [1], 5) fasting plasma glucose≥100 mg/dl (5.56 mmol/l) [20]. Subjects on drug therapy for hypertension, high blood glucose or abnormal lipid levels are also assigned to abnormal component status [20].

### Laboratory variables

A cobas 6000 multichannel analyzer (Hitachi High Technologies Corporation, Tokyo, Japan) and commercial kits (Roche Diagnostics, Mannheim, Germany) were used for measurement of cholesterol, HDL-C, triglycerides and glucose. Cholesterol (kit: CHOL2) and triglycerides (kit: TRIGL) were measured with an enzymatic colorimetric method. A homogeneous enzymatic colorimetric test was used for direct measurement of HDL-C (kit: HDLC3). LDL cholesterol (LDL-C) was calculated by the Friedewald formula. Glucose (kit: GLUC3) was measured by hexokinase method. Fasting insulin levels were measured with an electrochemiluminescence immunoassay (kit: Insulin) in a cobas e 411 analyzer (Hitachi High Technologies Corporation, Tokyo, Japan). Insulin resistance was estimated by Homeostasis Model Assessment (HOMA) equation. HOMA equals fasting serum insulin (μU/ml) times fasting plasma glucose (mmol/l) divided by 22.5 [21].

### Dietary intervention

The participants were free-living and were instructed not to alter their physical activity level during the study. Subjects visited Medical Nutrition Center three times: basal, at two weeks and at four weeks. The results of baseline and week-four visits are reported here. Subjects were asked to complete a three-day food intake record prior to each visit. The daily energy intake and the dietary composition were calculated from subjects' dietary history records by pen and paper [22]. Sample menus that were given to subjects were shown in the additional file 1. Women were instructed to follow a 1400 kcal 75-gram carbohydrate diet for four weeks. 21% of daily energy was from carbohydrate, 26% from protein and 53% from fat in the diet that was recommended to women. The analysis of the sample menu with BEBIS nutritional analysis software program (developed by Stuttgart-Hohenheim University, Stuttgart, Germany) showed that the prescribed diet for women included 52% polysaccharides, 17% disaccharides and 31% monosaccharides and 21 grams of fiber daily. The prescribed simple sugar intake constituted approximately 7% of the total energy intake. The distribution of fatty acids was as follows: 33% saturated, 17% polyunsaturated and 50% monounsaturated fatty acids. Ingested proteins were mainly of animal origin and vegetable proteins constituted approximately 22% of daily protein intake in the 1400 kcal 75-gram carbohydrate diet that was prescribed to women. Men were instructed to follow an 1800 kcal 100-gram carbohydrate diet for four weeks. 22% of daily energy was from carbohydrate, 29% from protein and 49% from fat in the diet that was recommended to men. The analysis of the sample menu with BEBIS nutritional analysis software program showed that the prescribed diet for men included 59% polysaccharides, 17% disaccharides and 24% monosaccharides and 27 grams of fiber daily. The prescribed simple sugar intake constituted approximately 5% of the total energy intake. The distribution of fatty acids was as follows: 32% saturated, 20% polyunsaturated and 48% monounsaturated fatty acids. Ingested proteins were mainly of animal origin and vegetable proteins constituted approximately 19% of daily protein intake in the 1800 kcal 100-gram carbohydrate diet that was prescribed to men. Meat, chicken or fish consumption was recommended once or twice per day to all subjects (additional file 1). As following a low-carbohydrate diet is expensive, subjects were given food products like salami, sausages, nuts and cheese to prevent noncompliance. Subjects were counseled to select from different food items at each visit. None of the participants consumed pork because pork is religiously forbidden in Islam and is not available on the Turkish market.

### Statistical methods

Before- and after-diet clinical and laboratory variables were compared. There was no control group. Last observation was carried forward for subjects who did not come to their last scheduled visit and for data that the investigators failed to collect or record. Results at week-2 were carried forward to week-4 for subjects who attended only to the baseline and week-2 visits. Subjects who attended to only baseline visit were excluded from data analysis. Categorical variables were presented as frequencies and percentages. Baseline social characteristics of men and women were compared with a χ^2 ^test. When there were any expected frequencies less than five, the rest of the categories were collapsed. Fisher's exact test was employed instead of χ^2 ^test when the expected frequencies were still less than five after collapsing the rest of the categories. Before- and after-diet results for continuous variables were presented as median, 25^th ^and 75^th ^percentile and were compared with a Wilcoxon signed ranks test. Before- and after-diet categorical variables were compared with a McNemar's test. The correlations between the change in energy intake, the change in carbohydrate intake or the change in insulin sensitivity and the change in outcome variables were found by calculating Pearson product-moment correlation coefficient or Spearman's rank correlation coefficient, as appropriate. The relation between starting weight and weight loss was estimated by Spearman's rank correlation coefficient. The Statistical Package for Social Sciences software, version 17.0 (SPSS Inc., Chicago, IL, USA) was used for statistical analyses.

## Results

**Figure 1:**
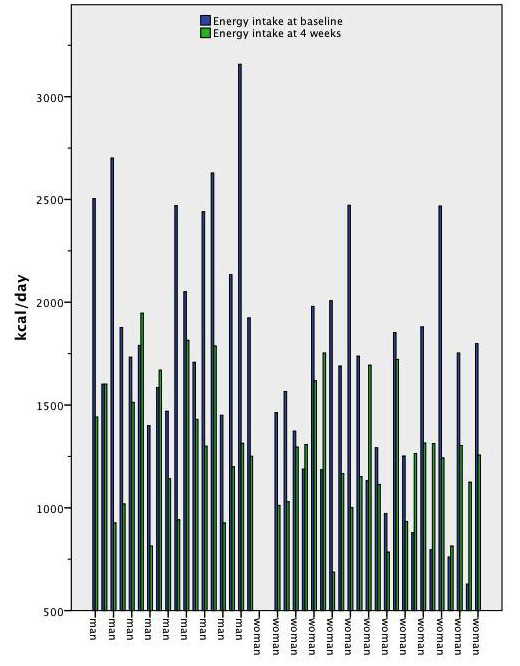
**Individual data of daily energy intake before and after low-carbohydrate diet**. Energy intake from food records before and after four weeks of an 1800 kcal 100-gram carbohydrate diet in overweight or obese men with low HDL-C levels and before and after four weeks of a 1400 kcal 75-gram carbohydrate diet in overweight or obese women with low HDL-C levels

**Figure 2:**
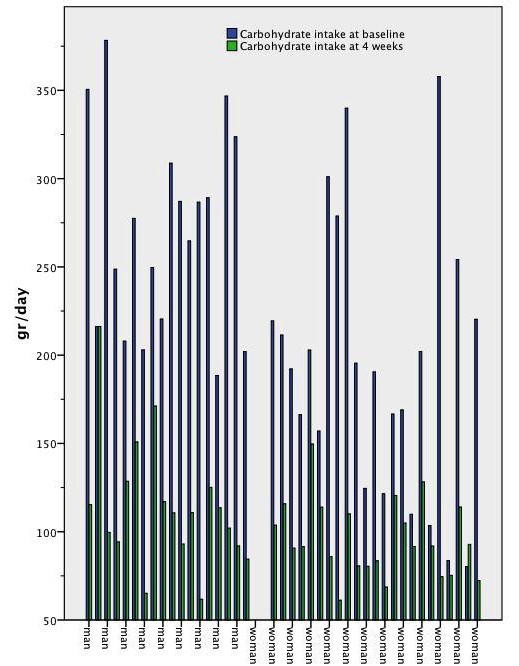
**Individual data of daily carbohydrate intake before and after low-carbohydrate diet**. Carbohydrate intake from food records before and after four weeks of an 1800 kcal 100-gram carbohydrate diet in overweight or obese men with low HDL-C levels and before and after four weeks of a 1400 kcal 75-gram carbohydrate diet in overweight or obese women with low HDL-C levels

There are 43 subjects, 18 men and 25 women in this study. Initially 50 subjects, 22 men and 28 women agreed to participate. Seven subjects (four men and three women) came only to the basal screening and did not follow the diet at all. Therefore, they were excluded from the analysis. Three subjects (one man and two women) came only to the baseline and week-2 visits and their results were carried forward to week-4. Eighty percent of (40 out of 50) subjects were compliant with the prescribed diet and attended to all three visits. Six men and six women were married to each other. The mean age of men was 38 and standard deviation (SD) was seven years. The mean age of women was 39 and SD was seven years. Social characteristics of the subjects were given in Table 1. Men were more educated than women. There were no differences in smoking, alcohol drinking and physical activity habits between men and women. Twelve men (67%) were overweight and six men (33%) were obese. Ten women (40%) were overweight and 15 women (60%) were obese (χ^2 ^= 2.978, p = 0.084 between men and women). Energy content and macronutrient composition before and after the diet were shown in Table 2. The median of men's baseline energy intake was 1901 kcal and was slightly higher than the energy value of the prescribed diet (1800 kcal). Compared to baseline, men achieved around 600 kcal energy deficit with the low-carbohydrate diet. There was a significant reduction in percent energy from carbohydrate and a significant increase in percent energy from protein and fat in men. Men's individual data for daily energy intake was illustrated in Figure 1. Men's individual data for daily carbohydrate intake was illustrated in Figure 2. Eighty-nine percent of men (16/18) had an energy intake below the target level of 1800 kcal at the end of the study. Only 39% of men (7/18) were able to achieve the target carbohydrate intake of 100 grams or less per day. The median of women's baseline energy intake was 1463 kcal and was close to the energy value of the prescribed diet (1400 kcal). Women had an insignificant 220 kcal energy deficit with the prescribed low-carbohydrate diet. There was a significant reduction in percent energy from carbohydrate and a significant increase in percent energy from fat in women. Percent energy from protein did not change significantly before and after the diet in women. Women's individual data for daily energy intake was illustrated in Figure 1. Women's individual data for daily carbohydrate intake was illustrated in Figure 2. Eighty-three percent of women (19/23) had an energy intake below the target level of 1400 kcal at the end of the study. Only 22% of women (5/23) were able to achieve the target carbohydrate intake of 75 grams or less per day.

**Table 1:** Social characteristics and life-style habits of subjects

Parameter	Men (n = 18)	Women (n = 25)	p value
Education			
Less than 6 years of education	39% (7)	72% (18)	0.030
Between 6 and 11 years of education*	22% (4)	20% (5)	1.000
More than 12 years of education*	39% (7)	8% (2)	0.023
Smoking			
Never-smoker	44% (8)	52% (13)	0.625
Ex-smoker*	22% (4)	4% (1)	0.144
Current-smoker	33% (6)	44% (11)	0.480
Alcohol drinking			
Non-drinker*	72% (13)	84% (21)	0.455
Drinker*	28% (5)	16% (4)	0.455
Exercise			
None to less than 1 hour/week	67% (12)	64% (16)	0.856
Between 1-4 hour/week*	22% (4)	20% (5)	1.000
More than 4 hour/week*	11% (2)	16% (4)	1.000

Non-drinker: subject does not consume any alcoholic beverage at all Drinker: consumes less than one glass of alcoholic beverage or more per week Percentage and frequency are given and χ^2 ^test is used to compare groups in unmarked rows *percentage and frequency are given and Fisher's exact test is used to compare groups

**Table 2:** Dietary energy and composition before and after four weeks of an energy-restricted low-carbohydrate diet in overweight or obese subjects with low HDL-C levels

	Before-diet*	After-diet*	p value†
Men (n = 18)‡			
Energy intake (kcal/day)	1901 (1598, 2479)	1307 (1000, 1619)	0.001
CHO intake (%)	55 (48, 61)	33 (28, 42)	<0.001
CHO intake (gram/day)	271 (214, 312)	111 (93, 126)	<0.001
Protein intake (%)	14 (13, 16)	22 (19, 26)	<0.001
Protein intake (gram/day)	63 (54, 99)	74 (54, 85)	0.619
Fat intake (%)	32 (27, 33)	45 (37, 47)	0.001
Fat intake (gram/day)	71 (51, 85)	65 (44, 80)	0.407
Women (n = 23)§			
Energy intake (kcal/day)	1463 (1132, 1852)	1243 (1012, 1313)	0.052
CHO intake (%)	53 (45, 58)	30 (28, 39)	<0.001
CHO intake (gram/day)	192 (125, 220)	92 (80, 114)	<0.001
Protein intake (%)	14 (12, 16)	18 (16, 21)	0.109
Protein intake (gram/day)	50 (33, 63)	53 (41, 69)	0.627
Fat intake (%)	31 (26, 36)	48 (43, 55)	<0.001
Fat intake (gram/day)	47 (41, 62)	68 (45, 79)	0.005

CHO: carbohydrate *median (25^th^, 75^th ^percentile) are given in the column †before- and after-diet results are compared with a Wilcoxon signed ranks test ‡men were prescribed an 1800 kcal 100-gram carbohydrate diet for four weeks §women were prescribed a 1400 kcal 75-gram carbohydrate diet for four weeks Daily macronutrient intake is given as percent energy from macronutrient in upper rows and as amount of macronutrient in grams in lower rows in lines illustrating macronutrient intake.

**Figure 3:**
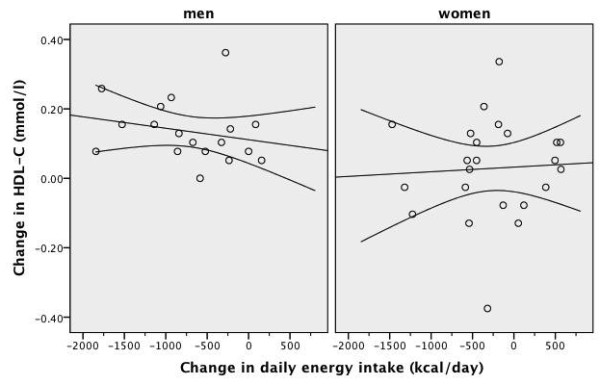
**Scatter plot of the change in daily energy intake and the change in HDL-C after four weeks of low-carbohydrate diet in overweight or obese subjects with low HDL-C levels**. A line of best fit with 95% confidence intervals is shown. Men were prescribed an 1800 kcal 100-gram carbohydrate diet for four weeks and women were prescribed a 1400 kcal 75-gram carbohydrate diet for four weeks. Change in HDL-C: HDL-C level at week-4 minus HDL-C level at baseline Change in daily energy intake: energy intake at week-4 minus energy intake at baseline

**Figure 4:**
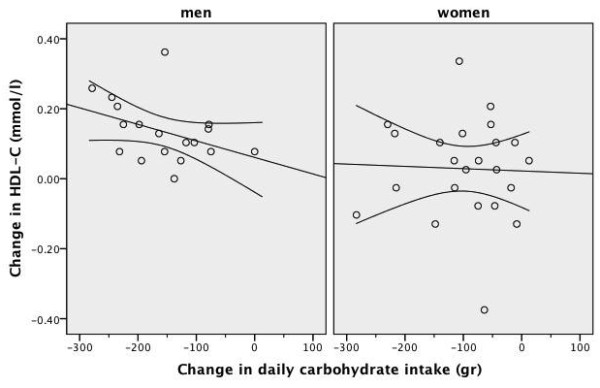
**Scatter plot of the change in daily carbohydrate intake and the change in HDL-C after four weeks of low-carbohydrate diet in overweight or obese subjects with low HDL-C levels**. A line of best fit with 95% confidence intervals is shown. Men were prescribed an 1800 kcal 100-gram carbohydrate diet for four weeks and women were prescribed a 1400 kcal 75-gram carbohydrate diet for four weeks. Change in HDL-C: HDL-C level at week-4 minus HDL-C level at baseline Change in daily carbohydrate intake: the amount of carbohydrate intake at week-4 minus the amount of carbohydrate intake at baseline

**Figure 5:**
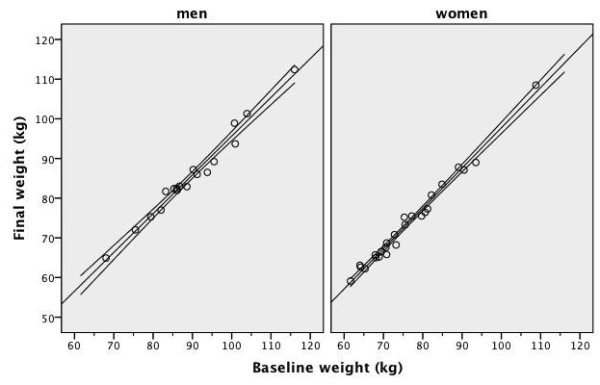
**Scatter plot of the baseline weight and the final weight after four weeks of low-carbohydrate diet in overweight or obese subjects with low HDL-C levels**. A line of best fit with 95% confidence intervals is shown. Men were prescribed an 1800 kcal 100-gram carbohydrate diet for four weeks and women were prescribed a 1400 kcal 75-gram carbohydrate diet for four weeks

**Figure 6:**
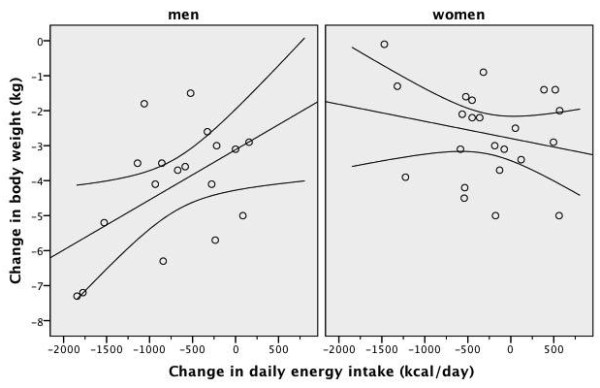
**Scatter plot of the change in daily energy intake and the change in body weight after four weeks of low-carbohydrate diet in overweight or obese subjects with low HDL-C levels**. A line of best fit with 95% confidence intervals is shown. Men were prescribed an 1800 kcal 100-gram carbohydrate diet for four weeks and women were prescribed a 1400 kcal 75-gram carbohydrate diet for four weeks. Change in body weight: weight at week-4 minus weight at baseline Change in daily energy intake: daily energy intake at week-4 minus daily energy intake at baseline

**Figure 7:**
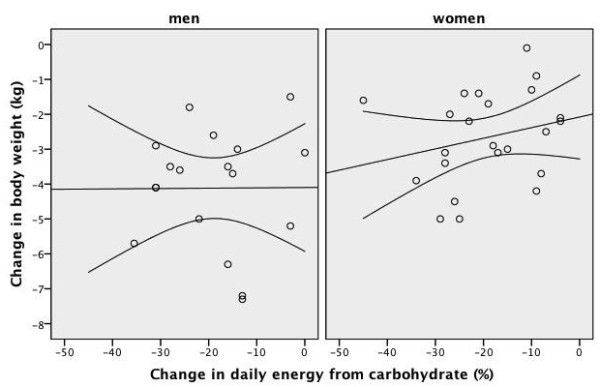
**Scatter plot of the change in daily energy intake from carbohydrate and the change in body weight after four weeks of low-carbohydrate diet in overweight or obese subjects with low HDL-C levels**. A line of best fit with 95% confidence intervals is shown. Men were prescribed an 1800 kcal 100-gram carbohydrate diet for four weeks and women were prescribed a 1400 kcal 75-gram carbohydrate diet for four weeks. Change in body weight: weight at week-4 minus weight at baseline Change in daily energy from carbohydrate: daily energy from carbohydrate at week-4 minus daily energy from carbohydrate at baseline

**Figure 8:**
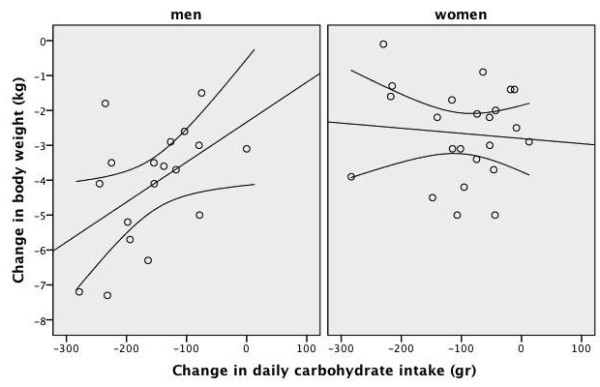
**Scatter plot of the change in daily carbohydrate intake and the change body weight after four weeks of low-carbohydrate diet in overweight or obese subjects with low HDL-C levels**. A line of best fit with 95% confidence intervals is shown. Men were prescribed an 1800 kcal 100-gram carbohydrate diet for four weeks and women were prescribed a 1400 kcal 75-gram carbohydrate diet for four weeks. Change in body weight: weight at week-4 minus weight at baseline Change in daily carbohydrate intake: the amount of carbohydrate intake at week-4 minus the amount of carbohydrate intake at baseline

As shown in Table 3 and Table 4, after four weeks of an energy-restricted low-carbohydrate diet, all subjects had significant reductions in body weight, BMI, waist circumference, body fat ratio, systolic blood pressure, and total cholesterol, triglyceride and insulin levels, TC/HDL-C ratio and HOMA value compared to baseline. There was a significant increase in median HDL-C levels in men, but not in women. The increase in HDL-C level was accompanied by a ~600 kcal daily energy deficit and a -4.7 kg difference in median body weight in men. After four weeks of a low-carbohydrate diet, there was no significant change in HDL-C levels in women. This was accompanied by an insignificant 220 kcal daily energy deficit but a significant -2.4 kg difference in median body weight in women. The frequency of the metabolic syndrome and abnormal HDL-C component significantly improved only in men and the frequency of the metabolic syndrome tended to improve in women (Table 5). The scatter plot of the change in daily energy intake versus the change in HDL-C level was presented in Figure 3. The scatter plot of the change in daily carbohydrate intake versus the change in HDL-C level was presented in Figure 4. The correlation coefficients between the change in energy intake, the change in carbohydrate intake or the change in insulin sensitivity and the change in outcome variables were weak (Table 6). The change in daily energy intake was significantly correlated with the change in triglycerides in men. The change in daily carbohydrate intake was significantly correlated with the change in body weight, total cholesterol/HDL-C ratio and triglycerides in the whole group. The change in daily carbohydrate intake was significantly correlated with the change in body weight, BMI, total cholesterol/HDL-C ratio and triglycerides in men and with the change in diastolic blood pressure, insulin levels and HOMA in women. There were more instances in which the change in carbohydrate intake was significantly correlated with outcome variables than the change in energy intake (Table 6). The correlation between baseline weight and weight loss was not significant, Spearman's rho= -0.186, p = 0.460 in men and Spearman's rho = 0.014, p = 0.948 in women. The starting weight and final weight were highly correlated (Spearman's rho = 0.983, p < 0.001 for men and Spearman's rho = 0.987, p < 0.001 for women). The relation between the baseline weight and the final weight was shown in Figure 5. The scatter plot of the change in daily energy intake versus weight loss was presented in Figure 6. The scatter plot of the change in daily energy intake from carbohydrate versus weight loss was presented in Figure 7. The scatter plot of the change in daily carbohydrate intake versus weight loss was presented in Figure 8.

**Table 3:** Anthropometric indices, bioimpedance analysis, blood pressure and laboratory variables before and after four weeks of an 1800 kcal 100-gram carbohydrate diet in overweight or obese men with low HDL-C levels

	n*	Before-diet†	After-diet†	p value‡
Body weight (kg)	18	87.7 (82.9, 96.8)	83.0 (80.5, 90.3)	<0.001
BMI (kg/m^2^)	18	29.1 (28.2, 30.6)	27.9 (26.8, 28.9)	<0.001
WC (cm)	16	104.8 (101.0, 108.1)	99.3 (95.4, 104.1)	<0.001
Body fat ratio (%)	17	25.4 (23.0, 27.7)	23.4 (20.2, 26.4)	0.010
Fat mass (kg)	17	22.0 (19.8, 24.9)	20.8 (16.6, 22.6)	<0.001
SBP (mmHg)	17	130 (123, 140)	124 (119, 134)	0.003
DBP (mmHg)	17	76 (73, 91)	80 (71, 84)	0.297
TC (mmol/l)	18	4.34 (4.13, 5.29)	4.20 (3.76, 4.95)	0.029
HDL-C (mmol/l)	18	0.83 (0.74, 0.88)	0.96 (0.82, 1.03)	<0.001
TC/HDL-C	18	5.56 (4.50, 7.28)	4.47 (3.91, 5.20)	<0.001
LDL-C (mmol/l)	18	3.11 (2.54, 3.76)	2.80 (2.32, 3.56)	0.058
TG (mmol/l)	18	1.23 (0.85, 1.85)	0.95 (0.63, 1.27)	0.004
Glucose (mmol/l)	18	4.94 (4.64, 5.13)	4.88 (4.66, 5.12)	0.983
Insulin (pmol/l)	17	80.3 (59.3, 98.3)	61.2 (45.5, 81.4)	0.005
HOMA	17	2.47 (1.74, 3.32)	1.89 (1.31, 2.63)	0.003

BMI: body mass index, WC: waist circumference, SBP: systolic blood pressure, DBP: diastolic blood pressure, TC: total cholesterol, HDL-C: high density lipoprotein cholesterol, LDL-C: low density lipoprotein cholesterol, TG: triglycerides, HOMA: homeostasis model assessment of insulin resistance *some data are missing for some of the variables †median (25^th^, 75^th ^percentile) are given in the column ‡before- and after-diet results are compared with a Wilcoxon signed ranks test

**Table 4:** Anthropometric indices, bioimpedance analysis, blood pressure and laboratory variables before and after four weeks of a 1400 kcal 75-gram carbohydrate diet in overweight or obese women with low HDL-C levels

	n*	Before-diet†	After-diet†	p value‡
Body weight (kg)	25	73.2 (68.5, 81.7)	70.8 (65.5, 79.1)	<0.001
BMI (kg/m^2^)	25	30.4 (28.0, 32.9)	29.5 (27.2, 31.4)	<0.001
WC (cm)	25	95.0 (87.5, 99.0)	92.0 (86.0, 96.5)	0.002
Body fat ratio (%)	24	38.0 (34.6, 39.7)	34.6 (32.7, 39.6)	<0.001
Fat mass (kg)	24	27.7 (23.4, 31.7)	24.3 (22.1, 29.9)	<0.001
SBP (mmHg)	25	123 (115, 132)	112 (107, 124)	0.005
DBP (mmHg)	25	82 (74, 91)	79 (76, 84)	0.253
TC (mmol/l)	25	4.50 (3.84, 5.25)	3.93 (3.56, 4.42)	<0.001
HDL-C (mmol/l)	25	1.03 (0.94, 1.09)	1.01 (0.89, 1.14)	0.165
TC/HDL-C	25	4.34 (3.73, 5.48)	3.81 (3.35, 4.30)	<0.001
LDL-C (mmol/l)	25	3.00 (2.37, 3.59)	2.46 (2.13, 2.93)	<0.001
TG (mmol/l)	25	1.12 (0.89, 1.58)	0.82 (0.73, 1.12)	0.011
Glucose (mmol/l)	25	5.11 (4.91, 5.41)	5.11 (4.83, 5.55)	0.742
Insulin (pmol/l)	23	61.0 (46.6, 96.4)	56.0 (38.0, 92.6)	0.005
HOMA	23	2.01 (1.44, 2.92)	1.71 (1.24, 2.74)	0.006

BMI: body mass index, WC: waist circumference, SBP: systolic blood pressure, DBP: diastolic blood pressure, TC: total cholesterol, HDL-C: high density lipoprotein cholesterol, LDL-C: low density lipoprotein cholesterol, TG: triglycerides, HOMA: homeostasis model assessment of insulin resistance *some data are missing for some of the variables †median (25^th^, 75^th ^percentile) are given in the column ‡before- and after-diet results are compared with a Wilcoxon signed ranks test

**Table 5:** The frequency of the metabolic syndrome and its components before and after four weeks of an energy-restricted low-carbohydrate diet in overweight or obese subjects with low HDL-C levels

	n*	Before-diet†	After-diet†	p value‡
Men§				
Metabolic Syndrome∥	16	50%	17%	0.031
Abnormal waist circumference criterion	16	56%	33%	0.125
Abnormal glucose criterion	18	11%	6%	1.000
Abnormal triglyceride criterion	18	33%	6%	0.063
Abnormal HDL-C criterion	18	100%	61%	0.016
Abnormal blood pressure criterion	17	53%	33%	0.250
Women¶				
Metabolic Syndrome∥	25	48%	24%	0.070
Abnormal waist circumference criterion	25	76%	60%	0.125
Abnormal glucose criterion	25	16%	28%	0.250
Abnormal triglyceride criterion	25	16%	4%	0.250
Abnormal HDL-C criterion	25	100%	88%	0.250
Abnormal blood pressure criterion	25	40%	24%	0.219

*some data are missing for some of the variables †percentage is given in the column ‡before- and after-diet frequencies are compared with a McNemar's test §men were prescribed an 1800 kcal 100-gram (22% of daily energy from) carbohydrate diet ∥the metabolic syndrome and its components are defined according to modified United States National Cholesterol Education Program Adult Treatment Panel III criteria [1,20] ¶women were prescribed a 1400 kcal 75-gram (21% of daily energy from) carbohydrate diet

**Table 6:** The correlation coefficients between the change in daily energy intake, the change in daily carbohydrate intake or the change in insulin sensitivity and outcome variables before and after four weeks of low-carbohydrate diet in overweight or obese subjects with low HDL-C levels

	Δ Energy intake	Δ CHO intake	Δ HOMA
All subjects†			
Δ body weight	0.286	0.321*	-0.085
Δ BMI	0.121	0.183	-0.133
Δ WC	0.132	0.142	-0.144
Δ body fat ratio	-0.182	-0.189	0.168
Δ fat mass	-0.120	0.103	0.131
Δ SBP	0.082	0.126	-0.060
Δ DBP	0.307	0.285	0.139
Δ TC	-0.084	-0.033	-0.030
Δ HDL-C	-0.172	-0.266	-0.059
Δ TC/HDL-C	0.134	0.319*	0.033
Δ LDL-C	-0.218	-0.214	-0.051
Δ TG	0.303	0.427**	0.059
Δ Glucose	-0.113	-0.84	0.589**
Δ Insulin	0.263	0.220	0.981**
Δ HOMA	0.213	0.162	1
Men‡§			
Δ body weight	0.386	0.493*	-0.530*
Δ BMI	0.352	0.482*	-0.569*
Δ WC	0.296	0.385	-0.300
Δ body fat ratio	0.088	0.211	0.414
Δ fat mass	0.115	0.303	0.072
Δ SBP	0.400	0.475	0.039
Δ DBP	0.352	0.126	0.196
Δ TC	0.185	0.375	0.012
Δ HDL-C	-0.349	-0.421	0.093
Δ TC/HDL-C	0.232	0.552*	-0.130
Δ LDL-C	-0.098	0.001	0.002
Δ TG	0.601**	0.677**	0.118
Δ Glucose	-0.209	-0.319	0.384
Δ Insulin	-0.034	-0.189	0.966**
Δ HOMA	-0.007	-0.174	1
Women‡∥			
Δ body weight	-0.123	0.001	0.160
Δ BMI	-0.149	-0.050	0.148
Δ WC	-0.140	-0.146	-0.186
Δ body fat ratio	-0.149	-0.179	0.115
Δ fat mass	-0.167	-0.131	0.180
Δ SBP	0.060	0.200	0.064
Δ DBP	0.211	0.442*	0.085
Δ TC	-0.038	-0.038	0.067
Δ HDL-C	0.057	-0.063	-0.036
Δ TC/HDL-C	-0.075	0.036	0.081
Δ LDL-C	-0.080	-0.137	0.083
Δ TG	0.029	0.260	0.106
Δ Glucose	-0.082	0.072	0.614**
Δ Insulin	0.394	0.449*	0.975**
Δ HOMA	0.390	0.435*	1

Δ: difference between the value at week-4 and the value at baseline, energy intake: daily energy intake obtained from food records, carbohydrate intake: daily amount of carbohydrate intake in grams obtained from food records, HOMA: homeostasis model assessment of insulin resistance, BMI: body mass index, WC: waist circumference, SBP: systolic blood pressure, DBP: diastolic blood pressure, TC: total cholesterol, HDL-C: high density lipoprotein cholesterol, LDL-C: low density lipoprotein cholesterol, TG: triglycerides, *p < 0.05 †Pearson product-moment correlation coefficient ‡Spearman's rank correlation coefficient §men were prescribed an 1800 kcal 100-gram (22% of daily energy from) carbohydrate diet ∥women were prescribed a 1400 kcal 75-gram (21% of daily energy from) carbohydrate diet

## Discussion

Beneficial effects of low-carbohydrate diets have been observed before. These studies were on normal weight normolipidemic subjects [17,18], overweight or obese subjects [13-16,23-25], subjects with hypertension [14,26], metabolic syndrome [14,16,26,27], hyperlipidemia [13,15] or diabetes [16,27,28]. Our study evaluates the effects of a low-carbohydrate diet in subjects with low HDL-C levels. Compared to North American or European populations, Turks have low levels of total cholesterol and HDL-C and relative roles of the metabolic syndrome and atherogenic dyslipidemia are more pronounced [29]. The mean and SD of HDL-C levels of Turkish men are 37 and 12 mg/dl, respectively. The mean and SD of HDL-C levels of Turkish women are 45 and 13 mg/dl, respectively [30]. Molecular genetic studies showed that single nucleotide polymorphisms in hepatic lipase, cholesterol ester transfer protein and ATP binding cassette transporter A1 genes are associated with plasma HDL-C levels in the Turkish population [31-34]. A previous study showed that 61% of adult nondiabetic Turkish population had low HDL-C levels (<40 mg/dl for men and <50 mg/dl for women) [35]. Therefore, we think that the effects of dietary therapy should be investigated in subjects with low HDL-C levels, especially in a population with a high prevalence of low HDL-C levels. Al-Sarraj and coworkers showed that a carbohydrate-restricted diet improves insulin resistance and all features of the metabolic syndrome except for the low HDL-C component in Emirati adults [36]. The authors commented that genetic predisposition to low HDL-C might explain the lack of effect of the low-carbohydrate diet on HDL-C levels [36]. Similarly, genetic factors have been found to be associated with low HDL-C levels in the Turkish population [31-34]. In contrast to the above-mentioned study in Emirati adults, a low-carbohydrate diet with energy restriction raised HDL-C levels in men in our study. As there are discrepant results among dietary intervention studies, further studies are needed in subjects with low HDL-C levels.

It has been reported that carbohydrate intake is related to HDL-C levels and every 100 gram/day increment of carbohydrate intake is associated with a 5.8 mg/dl (0.15 mmol/L) decrease in HDL-C levels after adjustment of confounders in a multivariate nutrient residual model [37]. A low-carbohydrate diet is effective in improving glycemic control and reducing hemoglobinA_1c _levels in subjects with diabetes mellitus [28]. Volek and Feinman proposed that the metabolic syndrome should be defined as an entity that favorably responds to carbohydrate restriction because a low-carbohydrate diet effectively targets each component of the metabolic syndrome: central obesity, low HDL-C, high triglycerides, high blood pressure and high blood glucose [10,11]. The mechanism of improvement is thought to be secondary to reduction of insulin resistance. Volek *et al*. stressed that dietary carbohydrate intake is a key element in disposal of excess dietary fat intake, directly or indirectly through the secretion of insulin [38]. Dietary carbohydrate modulates lipolysis, *de novo *lipogenesis and effects the interaction between dietary fat intake and plasma saturated fatty acid levels. Volek *et al*. showed that when carbohydrate intake was low and consequently plasma glucose and insulin levels were lower, a higher saturated fat intake would cause a reduction in relative proportions of circulating saturated fatty acids in triglyceride and cholesterol ester fractions [39]. In addition to improving classical cardiovascular risk factors, compared to a low-fat diet, a low-carbohydrate diet favorably effects nonclassical cardiovascular risk factors like apo B, apo A-I, apo B/apo A-I ratio, LDL particle size and distribution [39], postprandial lipemia and postprandial vascular endothelial function as assessed by flow-mediated dilatation [40]. Carbohydrate restriction also exerts an anti-inflammatory effect with a decrease in proinflammatory cytokine tumor necrosis factor-α, the chemokines interleukin-8 and monocyte chemotactic protein-1, the adhesion molecules E-selectin and intracellular adhesion molecule-1 and antifibrinolytic substance plasminogen-activator inhibitor-1 [41]. In summary, a large body of evidence indicates that abnormal fatty acid status and inflammatory state that are characteristics of the metabolic syndrome are better improved by a low-carbohydrate diet than a low-fat diet [38-41]. Low-carbohydrate diets have different favorable effects on CVD risk factors than low-fat diets [15,16]. Meckling and coworkers proposed that a low-fat diet should be initiated when reduction of blood cholesterol is the primary goal and a low-carbohydrate diet should be initiated when reduction of insulin resistance is the primary goal [13]. In view of our findings, we suggest that an energy-restricted low-carbohydrate diet should be initiated when an increase in HDL-C level is the primary goal.

### Limitations of the study

Even if some low-carbohydrate food items were provided free of charge, the vast majority of subjects could not achieve the target carbohydrate intake in our study. In contrast, most subjects reached their goal in caloric restriction. As low-carbohydrate food items are expensive, it may be difficult to follow a low-carbohydrate diet for the long-term in a developing country, like Turkey. Another limitation is that subjects were invited from a previous epidemiological study, not from the general population. The beneficial outcomes observed in this study were obtained with both energy and carbohydrate restriction. The results of this study should be viewed as pilot data that warrant further research. A long-term study with a control group is needed to validate our results. Studies that prescribe carbohydrate restriction with *ad libitum *energy intake show an increase in HDL-C levels with the dietary intervention [15,42-46], but there are few studies that show no significant change in HDL-C levels with *ad libitum *low-carbohydrate diets [16,27]. Studies that restricted both carbohydrate and energy intake showed a variable response in HDL-C levels as well. Recent selected dietary intervention studies that were published after 2002 and restricted carbohydrates to less than 40% of daily energy intake are summarized in Table 7. Male to female ratio, duration, the degree of energy and carbohydrate restriction, dietary composition and the amount of weight loss are highly variable in these studies. A consistent effect of a low-carbohydrate diet is a reduction of triglyceride levels. As seen in Table 7, studies of low-carbohydrate diets in which subjects lost greater amount of weight are consistently associated with an elevation of HDL-C levels whereas studies with smaller weight reduction are mostly associated with no significant change in HDL-C levels. Except for our study, none of the studies in Table 7 enrolled subjects only with low HDL-C levels. Frisch and coworkers showed no effect of a low-carbohydrate diet on HDL-C levels, although carbohydrate restriction was only 40% of daily energy intake in that study [12]. As seen in Table 7, only few studies showed an elevation of LDL-C levels with low-carbohydrate diets. We also did not observe an elevation of LDL-C level with a low-carbohydrate diet in our study. Carbohydrate restriction causes elevations in HDL-C levels from increased reverse cholesterol transport. The increase in HDL-C levels from carbohydrate restriction is characterized by an increase in HDL particle size but not quantity and an increase in lecithin:cholesterol acyltransferase activity with no change in cholesterol ester transfer protein activity [47].

**Table 7:** Results from recent selected studies* that evaluated the effects of low-carbohydrate diets

Author Year	n	**Basal BMI (kg/m**^**2**^**)**	Duration (week)	Δ Energy (kcal/day)	Dietary composition	Δ weight (kg)	HDL-C	TG	LDL-C	Reference
Sharman 2002	12 m	BMI<25	6	None	8/30/61	-2.2	↔	↓	↔	[18]
Volek 2003	10 w	BMI<25	4	None	10/29/60	-1.8	↑	↓	↑	[17]
Volek 2004	13 w	BMI≥25	4	-643	9/28/63	-3.0	↔	↔	↔	[25]
Sharman 2004	15 m	BMI≥25	6	-738	8/28/63	-6.1	↔	↓	↔	[24]
Meckling 2004	5 m/10 w	BMI≥25	10	-763	16/26/56	-7.0	↑	↓	↔	[13]
Noakes 2006	4 m/20 w	BMI>28	12	-615	25/30/50	-8.0	↑	↓	↑	[48]
Westman 2008†	7 m/14 w	BMI>27	24	-578	13/28/59	-11.1	↑	↓	↔	[28]
Brinkworth 2009	11 m/24 w	Abd obesity	52	-500	4/35/61	-14.5	↑	↓	↑	[49]
Jenkins 2009‡	10 m/15 w	BMI>27	4	-328	28/30/43	-3.9	↔	↓	↓	[50]
Volek 2009	10 m/10 w	BMI>25	12	-847	13/28/59	-10.1	↑	↓	↔	[39]
Can 2010	18 m	BMI≥25	4	-600	33/22/45	-3.7	↑	↓	↔	T
Can 2010	25 w	BMI≥25	4	-220	30/18/48	-1.1	↔	↓	↓	T

BMI: body mass index, Δ Energy: difference between end-study and baseline mean or median daily energy intake, Dietary composition: percentage of daily energy from carbohydrate/protein/fat, Δ weight: difference between mean or median end-study and baseline weight, HDL-C: high density lipoprotein cholesterol, TG: triglycerides, LDL-C: low density lipoprotein cholesterol, m: men, w: women, Abd obesity: abdominal obesity, T: this study. *selected studies published after 2002 and restricted carbohydrate intake to less than 40% of daily energy intake are included in the table. A summary of studies published prior to 2004 can be found in the review by Volek and Feinman [10]. Studies of low-carbohydrate diets based on *ad libitum *energy intake, glycemic index or glycemic load are excluded from the table. †only subjects with diabetes mellitus were enrolled into the study ‡protein and fat are from plant origin in the study

## Conclusions

A short term energy-restricted low-carbohydrate diet causes weight loss, fat loss, a decrease in total cholesterol and triglyceride levels and an improvement in insulin resistance in overweight and obese subjects with low HDL-C levels. HDL-C levels increased significantly with energy restriction, carbohydrate restriction and weight loss in men. HDL-C levels did not change in women in whom there was no significant energy restriction but a significant carbohydrate restriction and a relatively small but significant amount of weight loss. Differences in gender, baseline characteristics of subjects and the dietary intervention may account for divergent outcomes in HDL-C levels between men and women. The results of this study suggest that both energy and carbohydrate restriction should be considered in overweight and obese subjects with low HDL-C levels, especially when LDL-C levels are not elevated.

## Abbreviations

Abd obesity: abdominal obesity; BMI: body mass index; CVD: cardiovascular disease; DBP: diastolic blood pressure; HDL-C: high density lipoprotein cholesterol; HOMA: homeostasis model assessment of insulin resistance; LDL-C: low density lipoprotein cholesterol; m: men; NCEP: The United States National Cholesterol Education Program Adult Treatment Panel III; SBP: systolic blood pressure; SD: standard deviation; T: this study; TC: total cholesterol; TG: triglycerides; w: women; WC: waist circumference.

## Competing interests

The authors declare that they have no competing interests.

## Authors' contributions

ASC planned the study, obtained Ethics Committee approval, performed medical examinations and wrote the manuscript. CU obtained anthropometric indices, evaluated dietary intake records and gave the dietary instructions to subjects. KEP measured laboratory variables. All authors read and approved the final manuscript.

## Pre-publication history

The pre-publication history for this paper can be accessed here:

http://www.biomedcentral.com/1472-6823/10/18/prepub

## Supplementary Material

Additional file 1 **Sample menus**. Sample menus for an 1800 kcal 100-gram carbohydrate diet and a 1400 kcal 75-gram carbohydrate diet. Click here for file

## References

[B1] Executive Summary of The Third Report of The National Cholesterol Education Program (NCEP) Expert Panel on Detection, Evaluation, And Treatment of High Blood Cholesterol In Adults (Adult Treatment Panel III) Jama 2001 285 2486 2497 10.1001/jama.285.19.2486 11368702

[B2] deGoma EM Leeper NJ Heidenreich PA Clinical significance of high-density lipoprotein cholesterol in patients with low low-density lipoprotein cholesterol J Am Coll Cardiol 2008 51 49 55 10.1016/j.jacc.2007.07.086 18174036

[B3] Grover SA Kaouache M Joseph L Barter P Davignon J Evaluating the incremental benefits of raising high-density lipoprotein cholesterol levels during lipid therapy after adjustment for the reductions in other blood lipid levels Arch Intern Med 2009 169 1775 1780 10.1001/archinternmed.2009.328 19858435

[B4] Katcher HI Hill AM Lanford JL Yoo JS Kris-Etherton PM Lifestyle approaches and dietary strategies to lower LDL-cholesterol and triglycerides and raise HDL-cholesterol Endocrinol Metab Clin North Am 2009 38 45 78 10.1016/j.ecl.2008.11.010 19217512

[B5] Perez-Lopez FR Chedraui P Haya J Cuadros JL Effects of the Mediterranean diet on longevity and age-related morbid conditions Maturitas 2009 64 67 79 10.1016/j.maturitas.2009.07.013 19720479

[B6] Rumawas ME Meigs JB Dwyer JT McKeown NM Jacques PF Mediterranean-style dietary pattern, reduced risk of metabolic syndrome traits, and incidence in the Framingham Offspring Cohort Am J Clin Nutr 2009 90 1608 1614 10.3945/ajcn.2009.27908 19828705 PMC3152203

[B7] Decewicz DJ Neatrour DM Burke A Haberkorn MJ Patney HL Vernalis MN Ellsworth DL Effects of cardiovascular lifestyle change on lipoprotein subclass profiles defined by nuclear magnetic resonance spectroscopy Lipids Health Dis 2009 8 26 10.1186/1476-511X-8-26 19563671 PMC2713234

[B8] Lichtenstein AH Appel LJ Brands M Carnethon M Daniels S Franch HA Franklin B Kris-Etherton P Harris WS Howard B Diet and lifestyle recommendations revision 2006: a scientific statement from the American Heart Association Nutrition Committee Circulation 2006 114 82 96 10.1161/CIRCULATIONAHA.106.176158 16785338

[B9] WHO Obesity: preventing and managing the global epidemic. Report of a WHO consultation on obesity World Health Organization Technical Report Series WHO/NUT/NCD/981 1997 Geneva: WHO 11234459

[B10] Volek JS Feinman RD Carbohydrate restriction improves the features of Metabolic Syndrome. Metabolic Syndrome may be defined by the response to carbohydrate restriction Nutr Metab (Lond) 2005 2 31 10.1186/1743-7075-2-31 16288655 PMC1323303

[B11] Feinman RD Volek JS Carbohydrate restriction as the default treatment for type 2 diabetes and metabolic syndrome Scand Cardiovasc J 2008 42 256 263 10.1080/14017430802014838 18609058

[B12] Frisch S Zittermann A Berthold HK Gotting C Kuhn J Kleesiek K Stehle P Kortke H A randomized controlled trial on the efficacy of carbohydrate-reduced or fat-reduced diets in patients attending a telemedically guided weight loss program Cardiovasc Diabetol 2009 8 36 10.1186/1475-2840-8-36 19615091 PMC2722581

[B13] Meckling KA O'Sullivan C Saari D Comparison of a low-fat diet to a low-carbohydrate diet on weight loss, body composition, and risk factors for diabetes and cardiovascular disease in free-living, overweight men and women J Clin Endocrinol Metab 2004 89 2717 2723 10.1210/jc.2003-031606 15181047

[B14] Tay J Brinkworth GD Noakes M Keogh J Clifton PM Metabolic effects of weight loss on a very-low-carbohydrate diet compared with an isocaloric high-carbohydrate diet in abdominally obese subjects J Am Coll Cardiol 2008 51 59 67 10.1016/j.jacc.2007.08.050 18174038

[B15] Yancy WS Jr Olsen MK Guyton JR Bakst RP Westman EC A low-carbohydrate, ketogenic diet versus a low-fat diet to treat obesity and hyperlipidemia: a randomized, controlled trial Ann Intern Med 2004 140 769 777 15148063 10.7326/0003-4819-140-10-200405180-00006

[B16] Stern L Iqbal N Seshadri P Chicano KL Daily DA McGrory J Williams M Gracely EJ Samaha FF The effects of low-carbohydrate versus conventional weight loss diets in severely obese adults: one-year follow-up of a randomized trial Ann Intern Med 2004 140 778 785 15148064 10.7326/0003-4819-140-10-200405180-00007

[B17] Volek JS Sharman MJ Gomez AL Scheett TP Kraemer WJ An isoenergetic very low carbohydrate diet improves serum HDL cholesterol and triacylglycerol concentrations, the total cholesterol to HDL cholesterol ratio and postprandial lipemic responses compared with a low fat diet in normal weight, normolipidemic women J Nutr 2003 133 2756 2761 12949361 10.1093/jn/133.9.2756

[B18] Sharman MJ Kraemer WJ Love DM Avery NG Gomez AL Scheett TP Volek JS A ketogenic diet favorably affects serum biomarkers for cardiovascular disease in normal-weight men J Nutr 2002 132 1879 1885 12097663 10.1093/jn/132.7.1879

[B19] Mahley RW Can S Ozbayrakci S Bersot TP Tanir S Palaoglu KE Pepin GM Modulation of high-density lipoproteins in a population in Istanbul, Turkey, with low levels of high-density lipoproteins Am J Cardiol 2005 96 547 555 10.1016/j.amjcard.2005.04.018 16098310

[B20] Grundy SM Cleeman JI Daniels SR Donato KA Eckel RH Franklin BA Gordon DJ Krauss RM Savage PJ Smith SC Jr Diagnosis and management of the metabolic syndrome: an American Heart Association/National Heart, Lung, and Blood Institute Scientific Statement Circulation 2005 112 2735 2752 10.1161/CIRCULATIONAHA.105.169404 16157765

[B21] Matthews DR Hosker JP Rudenski AS Naylor BA Treacher DF Turner RC Homeostasis model assessment: insulin resistance and beta-cell function from fasting plasma glucose and insulin concentrations in man Diabetologia 1985 28 412 419 10.1007/BF00280883 3899825

[B22] Food Exchange List http://www.nhlbi.nih.gov/health/public/heart/obesity/lose_wt/fd_exch.htm

[B23] Sacks FM Bray GA Carey VJ Smith SR Ryan DH Anton SD McManus K Champagne CM Bishop LM Laranjo N Comparison of weight-loss diets with different compositions of fat, protein, and carbohydrates N Engl J Med 2009 360 859 873 10.1056/NEJMoa0804748 19246357 PMC2763382

[B24] Sharman MJ Gomez AL Kraemer WJ Volek JS Very low-carbohydrate and low-fat diets affect fasting lipids and postprandial lipemia differently in overweight men J Nutr 2004 134 880 885 15051841 10.1093/jn/134.4.880

[B25] Volek JS Sharman MJ Gomez AL DiPasquale C Roti M Pumerantz A Kraemer WJ Comparison of a very low-carbohydrate and low-fat diet on fasting lipids, LDL subclasses, insulin resistance, and postprandial lipemic responses in overweight women J Am Coll Nutr 2004 23 177 184 15047685 10.1080/07315724.2004.10719359

[B26] Muzio F Mondazzi L Harris WS Sommariva D Branchi A Effects of moderate variations in the macronutrient content of the diet on cardiovascular disease risk factors in obese patients with the metabolic syndrome Am J Clin Nutr 2007 86 946 951 17921369 10.1093/ajcn/86.4.946

[B27] Seshadri P Iqbal N Stern L Williams M Chicano KL Daily DA McGrory J Gracely EJ Rader DJ Samaha FF A randomized study comparing the effects of a low-carbohydrate diet and a conventional diet on lipoprotein subfractions and C-reactive protein levels in patients with severe obesity Am J Med 2004 117 398 405 10.1016/j.amjmed.2004.04.009 15380496

[B28] Westman EC Yancy WS Jr Mavropoulos JC Marquart M McDuffie JR The effect of a low-carbohydrate, ketogenic diet versus a low-glycemic index diet on glycemic control in type 2 diabetes mellitus Nutr Metab (Lond) 2008 5 36 10.1186/1743-7075-5-36 19099589 PMC2633336

[B29] Onat A Ceyhan K Basar O Erer B Toprak S Sansoy V Metabolic syndrome: major impact on coronary risk in a population with low cholesterol levels--a prospective and cross-sectional evaluation Atherosclerosis 2002 165 285 292 10.1016/S0021-9150(02)00236-8 12417279

[B30] Onat A Risk factors and cardiovascular disease in Turkey Atherosclerosis 2001 156 1 10 10.1016/S0021-9150(01)00500-7 11368991

[B31] Ozsait B Komurcu Bayrak E Poda M Can G Hergenc G Onat A Humphries SE Erginel Unaltuna N CETP TaqIB polymorphism in Turkish adults: association with dyslipidemia and metabolic syndrome Anadolu Kardiyol Derg 2008 8 324 330 18849221

[B32] Hodoglugil U Williamson DW Mahley RW Polymorphisms in the hepatic lipase gene affect plasma HDL-cholesterol levels in a Turkish population J Lipid Res 2010 51 422 430 10.1194/jlr.P001578 19734193 PMC2803245

[B33] Dogru-Abbasoglu S Parildar-Karpuzoglu H Depboylu B Cine N Uysal M Aykac-Toker G I405V and TaqIB polymorphisms of the cholesteryl ester transfer protein and their relation to serum lipid and lipoprotein levels in a Turkish population Cell Biochem Funct 2009 27 76 80 10.1002/cbf.1536 19165812

[B34] Hodoglugil U Williamson DW Huang Y Mahley RW Common polymorphisms of ATP binding cassette transporter A1, including a functional promoter polymorphism, associated with plasma high density lipoprotein cholesterol levels in Turks Atherosclerosis 2005 183 199 212 10.1016/j.atherosclerosis.2005.03.004 15935359

[B35] Can AS Bersot TP Analysis of agreement among definitions of metabolic syndrome in nondiabetic Turkish adults: a methodological study BMC Public Health 2007 7 353 10.1186/1471-2458-7-353 18088443 PMC2249584

[B36] Al-Sarraj T Saadi H Calle MC Volek JS Fernandez ML Carbohydrate restriction, as a first-line dietary intervention, effectively reduces biomarkers of metabolic syndrome in Emirati adults J Nutr 2009 139 1667 1676 10.3945/jn.109.109603 19587123

[B37] Merchant AT Anand SS Kelemen LE Vuksan V Jacobs R Davis B Teo K Yusuf S Carbohydrate intake and HDL in a multiethnic population Am J Clin Nutr 2007 85 225 230 17209200 10.1093/ajcn/85.1.225

[B38] Volek JS Fernandez ML Feinman RD Phinney SD Dietary carbohydrate restriction induces a unique metabolic state positively affecting atherogenic dyslipidemia, fatty acid partitioning, and metabolic syndrome Prog Lipid Res 2008 47 307 318 10.1016/j.plipres.2008.02.003 18396172

[B39] Volek JS Phinney SD Forsythe CE Quann EE Wood RJ Puglisi MJ Kraemer WJ Bibus DM Fernandez ML Feinman RD Carbohydrate restriction has a more favorable impact on the metabolic syndrome than a low fat diet Lipids 2009 44 297 309 10.1007/s11745-008-3274-2 19082851

[B40] Volek JS Ballard KD Silvestre R Judelson DA Quann EE Forsythe CE Fernandez ML Kraemer WJ Effects of dietary carbohydrate restriction versus low-fat diet on flow-mediated dilation Metabolism 2009 58 1769 1777 10.1016/j.metabol.2009.06.005 19632695

[B41] Forsythe CE Phinney SD Fernandez ML Quann EE Wood RJ Bibus DM Kraemer WJ Feinman RD Volek JS Comparison of low fat and low carbohydrate diets on circulating fatty acid composition and markers of inflammation Lipids 2008 43 65 77 10.1007/s11745-007-3132-7 18046594

[B42] McAuley KA Hopkins CM Smith KJ McLay RT Williams SM Taylor RW Mann JI Comparison of high-fat and high-protein diets with a high-carbohydrate diet in insulin-resistant obese women Diabetologia 2005 48 8 16 10.1007/s00125-004-1603-4 15616799

[B43] Dansinger ML Gleason JA Griffith JL Selker HP Schaefer EJ Comparison of the Atkins, Ornish, Weight Watchers, and Zone diets for weight loss and heart disease risk reduction: a randomized trial Jama 2005 293 43 53 10.1001/jama.293.1.43 15632335

[B44] Brehm BJ Seeley RJ Daniels SR D'Alessio DA A randomized trial comparing a very low carbohydrate diet and a calorie-restricted low fat diet on body weight and cardiovascular risk factors in healthy women J Clin Endocrinol Metab 2003 88 1617 1623 10.1210/jc.2002-021480 12679447

[B45] Gardner CD Kiazand A Alhassan S Kim S Stafford RS Balise RR Kraemer HC King AC Comparison of the Atkins, Zone, Ornish, and LEARN diets for change in weight and related risk factors among overweight premenopausal women: the A TO Z Weight Loss Study: a randomized trial Jama 2007 297 969 977 10.1001/jama.297.9.969 17341711

[B46] Foster GD Wyatt HR Hill JO McGuckin BG Brill C Mohammed BS Szapary PO Rader DJ Edman JS Klein S A randomized trial of a low-carbohydrate diet for obesity N Engl J Med 2003 348 2082 2090 10.1056/NEJMoa022207 12761365

[B47] Wood RJ Effect of dietary carbohydrate restriction with and without weight loss on atherogenic dyslipidemia Nutr Rev 2006 64 539 545 10.1111/j.1753-4887.2006.tb00187.x 17274496

[B48] Noakes M Foster PR Keogh JB James AP Mamo JC Clifton PM Comparison of isocaloric very low carbohydrate/high saturated fat and high carbohydrate/low saturated fat diets on body composition and cardiovascular risk Nutr Metab (Lond) 2006 3 7 10.1186/1743-7075-3-7 16403234 PMC1368980

[B49] Brinkworth GD Noakes M Buckley JD Keogh JB Clifton PM Long-term effects of a very-low-carbohydrate weight loss diet compared with an isocaloric low-fat diet after 12 mo Am J Clin Nutr 2009 90 23 32 10.3945/ajcn.2008.27326 19439458

[B50] Jenkins DJ Wong JM Kendall CW Esfahani A Ng VW Leong TC Faulkner DA Vidgen E Greaves KA Paul G Singer W The effect of a plant-based low-carbohydrate ("Eco-Atkins") diet on body weight and blood lipid concentrations in hyperlipidemic subjects Arch Intern Med 2009 169 1046 1054 10.1001/archinternmed.2009.115 19506174

